# Supplementary figures and images for: The Diversity and Functional Capacity of Microbes Associated with Coastal Macrophytes
Source: mSystems. 2022 Aug 22;7(5):e00592-22. doi: 10.1128/msystems.00592-22 (PMC9601103; doi:10.1128/msystems.00592-22)

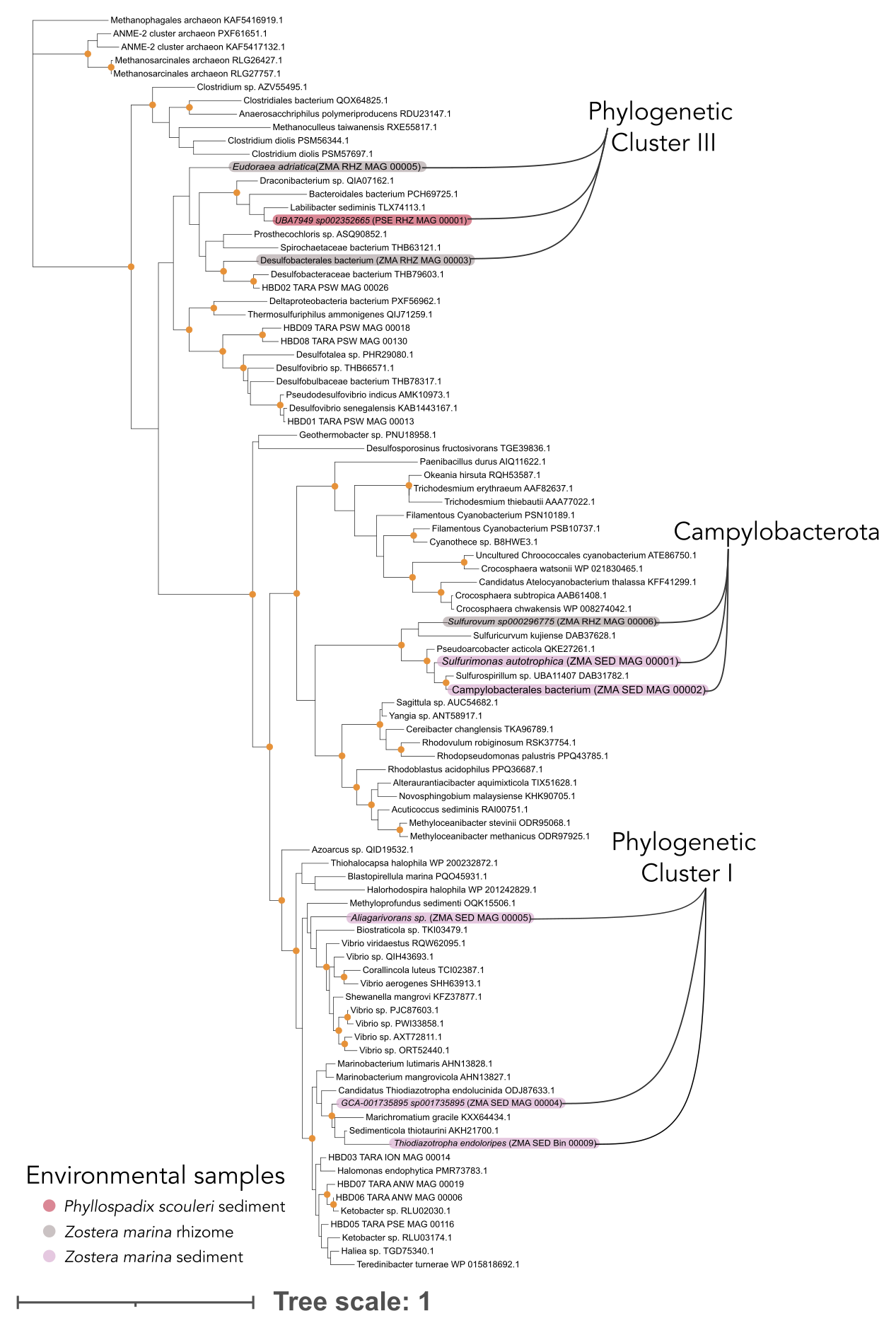

Supplement: FIG S3 [file msystems.00592-22-sf003.tiff]

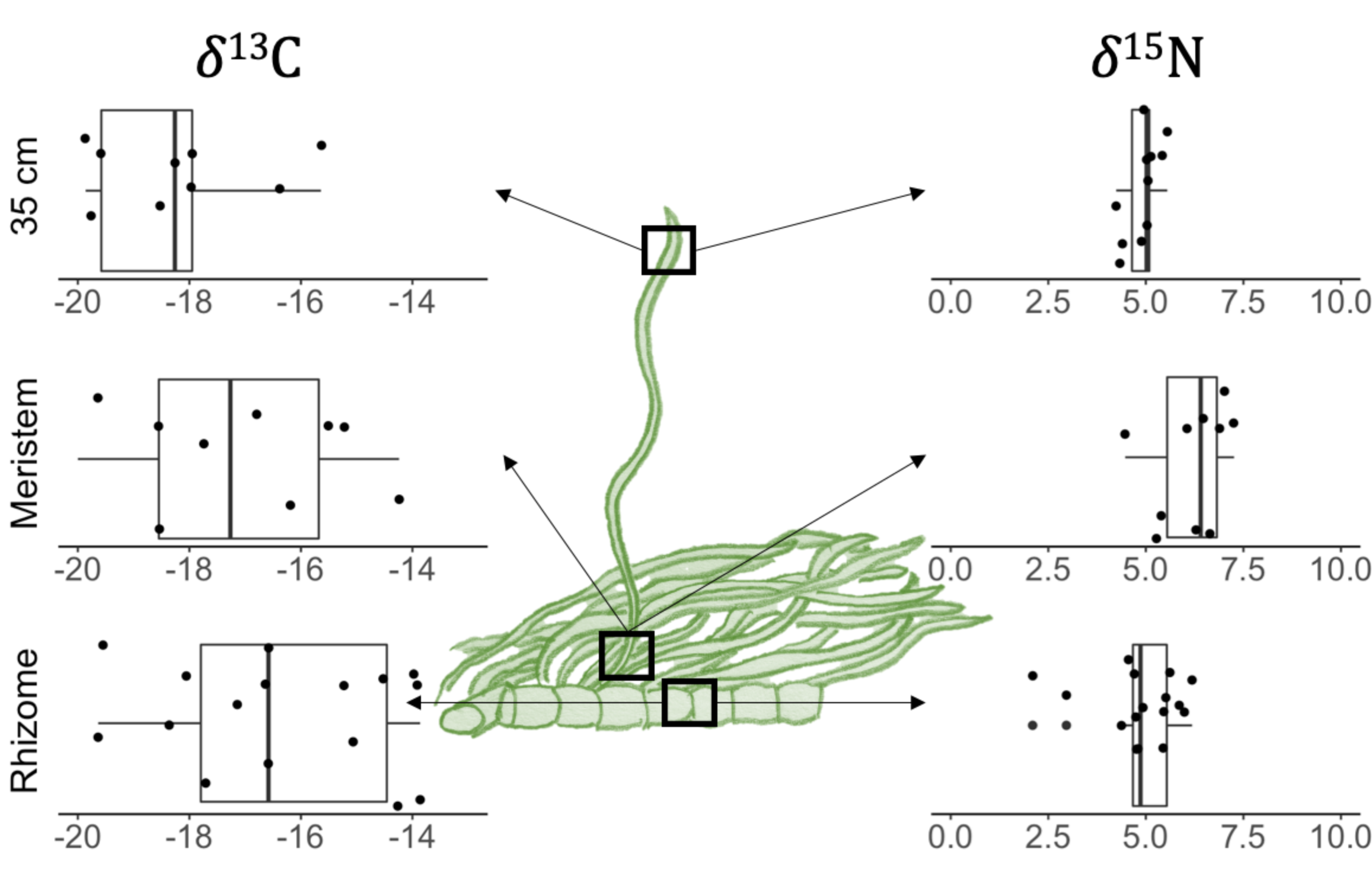

Supplement: FIG S1 [file msystems.00592-22-sf001.tiff]
