# Supplementary material for: The Diversity and Functional Capacity of Microbes Associated with Coastal Macrophytes
Source: mSystems. 2022 Aug 22;7(5):e00592-22. doi: 10.1128/msystems.00592-22 (PMC9601103; doi:10.1128/msystems.00592-22)
Supplement: TEXT S1 [file msystems.00592-22-s0001.docx]

**Appendix S1: Additional Methods**

**Quantification of carbon and nitrogen stable isotopes in *P. scouleri***

The δ15N and δ13C values were assayed from collected surfgrass samples. Surfgrass blades were sampled in two locations (n = 12): the basal meristematic region just distal to the sheath and on the blade 35 cm above the rhizome. Additionally, we collected rhizome and blade tissue (n = 16) the following year (2017) from nearby patches in order to study whether nitrogen-fixation was occurring in the surfgrass rhizome. Tissue samples were placed in aluminum foil envelopes, dried at 50°C for 48 h, ground to a fine powder with a 3 mm stainless steel ball in a Genogrinder (Spex Sample Prep., Metuchen, New Jersey, USA) and packed into a 3.5 mm tin capsule prior to elemental and isotopic analysis. Samples were analyzed using a Costech 4010 Elemental Analyzer combustion system (Costech, Valencia, California, USA) coupled to a Thermo DeltaV Plus IRMS (Thermo Fisher Scientific, Waltham, Massachusetts, USA) via a Thermo Conflo IV interface at the University of Chicago or at Northwestern University. The reproducibility was 0.11% for δ13C and 0.17% for δ15N at the University of Chicago lab.
